# Structure and allostery in major facilitator superfamily symport mechanisms

**DOI:** 10.1107/S2059798326007485

**Published:** 2026-08-21

**Authors:** Lan Guan

**Affiliations:** a Department of Cell Physiology and Molecular Biophysics, Center for Membrane Protein Research, School of Medicine, Texas Tech University Health Sciences Center, Lubbock, Texas, USA; University of Regensburg, Germany

**Keywords:** cryoEM, X-ray crystallography, HDX-MS, nanobodies, melibiose/Na^+^ symport, conformational changes, MFS transporters, H^+^-coupled symport, H^+^-coupled antiporters

## Abstract

The solute carriers of the major facilitator superfamily (MFS) play significant roles in human health and disease. The melibiose transporter of *Salmonella enterica* serovar Typhimurium (MelB_St_) catalyzes the stoichiometric symport of galactosides with Na^+^, H^+^ or Li^+^ and is a well studied prototype of MFS transporters. With a large body of data from extensive functional analyses using multiple transport and binding assays, structural characterizations of multiple states by X-ray crystallography and cryoEM single-particle analysis, and dynamic elucidations using hydrogen–deuterium exchange mass spectrometry (HDX-MS), the molecular recognition of the primary substrate galactoside and its coupling cation Na^+^, H^+^ or Li^+^ has been elucidated at the molecular level. Cooperative binding of both solutes is recognized as the core symport mechanism that supports the transporter’s functions more effectively in a sugar-scarce environment. Structural and dynamic studies show that conformational dynamics significantly influence sugar binding but have minimal effect on Na^+^. Na^+^ acts as an allosteric activator, increasing sugar affinity by stabilizing the inner barrier and constraining conformational flexibility. All data are consistent with the previously constructed stepped-binding kinetic model for melibiose symport with Na^+^; in addition, MFS uniport and antiport mechanisms are discussed.

## Abstract 2

Conformational dynamics in the melibiose/Na^+^ cotransporter MelB significantly influence the binding of the primary sugar substrate but have minimal effect on the co-substrate Na^+^. Na^+^ acts as an allosteric activator, increasing sugar affinity by stabilizing the inner barrier and constraining conformational flexibility.

## 1. Introduction

The major facilitator superfamily (MFS) of transporters, the major members of the solute carrier (SLC) family of transporters (Pao *et al.*, 1998 ▸), comprises a large number of facilitators (uniporters) and secondary active transporters (symporters or antiporters). MFS transporters enable the uptake of diverse solutes across cell membranes, serving crucial functions in physiology, disease processes and drug absorption and distribution, and are increasingly recognized as potential drug targets. Recent rapid advances in membrane-protein research have greatly enhanced our understanding of protein conformation and transport mechanisms (Guan & Kaback, 2006 ▸; Drew *et al.*, 2021 ▸; Guan, 2023 ▸). This review will focus on the most recent advances in the Na^+^-coupled melibiose transporter of *Salmonella enterica* serovar Typhimurium (MelB_St_), a well characterized representative for understanding the transport mechanisms, including both uniport and symport, mediated by MFS transporters.

MelB_St_ catalyzes the stoichiometric symport of galactopyranoside with Na^+^, H^+^ or Li^+^ (Wilson & Ding, 2001 ▸; Meyer-Lipp *et al.*, 2006 ▸; Guan *et al.*, 2011 ▸; Granell *et al.*, 2010 ▸; Guan, 2018 ▸; Guan & Hariharan, 2021 ▸; Hariharan, Shi *et al.*, 2024 ▸; Hariharan, Bakhtiiari *et al.*, 2024 ▸). In the past, mutagenesis studies, including cysteine-scanning and site-directed mutagenesis, in combination with a battery of biochemical and biophysical analyses, have provided fundamental insights into the binding of the primary substrate, galactosides, and the co-substrate, the coupling cation Na^+^, H^+^ or Li^+^, as well as their positive cooperativity (Markham *et al.*, 2021 ▸; Katsube *et al.*, 2022 ▸; Hariharan & Guan, 2017 ▸, 2021 ▸; Guan & Hariharan, 2021 ▸). Thus, binding of one substrate increases the affinity of MelB_St_ for the other, and by this mechanism Na^+^ significantly increases the binding affinity for sugar, allowing the transporter to function efficiently in a sugar-scarce environment. Recent comprehensive structural and dynamic investigations utilizing X-ray crystallography (Guan & Hariharan, 2021 ▸; Hariharan *et al.*, 2026 ▸), cryo-electron microscopy (cryo-EM) single-particle analysis assisted by nanobody and nanodiscs (Hariharan, Shi *et al.*, 2024 ▸; Katsube *et al.*, 2023 ▸; Hariharan & Guan, 2024 ▸), hydrogen/deuterium exchange-mass spectrometry (HDX-MS; Hariharan *et al.*, 2026 ▸) and molecular-dynamics (MD) simulations (Katsube *et al.*, 2022 ▸; Hariharan, Bakhtiiari *et al.*, 2024 ▸; Liang & Guan, 2024 ▸) have provided further support for the molecular basis of cooperative binding and transport mechanisms. Moreover, these studies explained how conformational changes govern substrate binding and how Na^+^ and the sugar substrate modulate conformational dynamics.

## 2. Structural studies of MelB_St_

**Figure 1:**
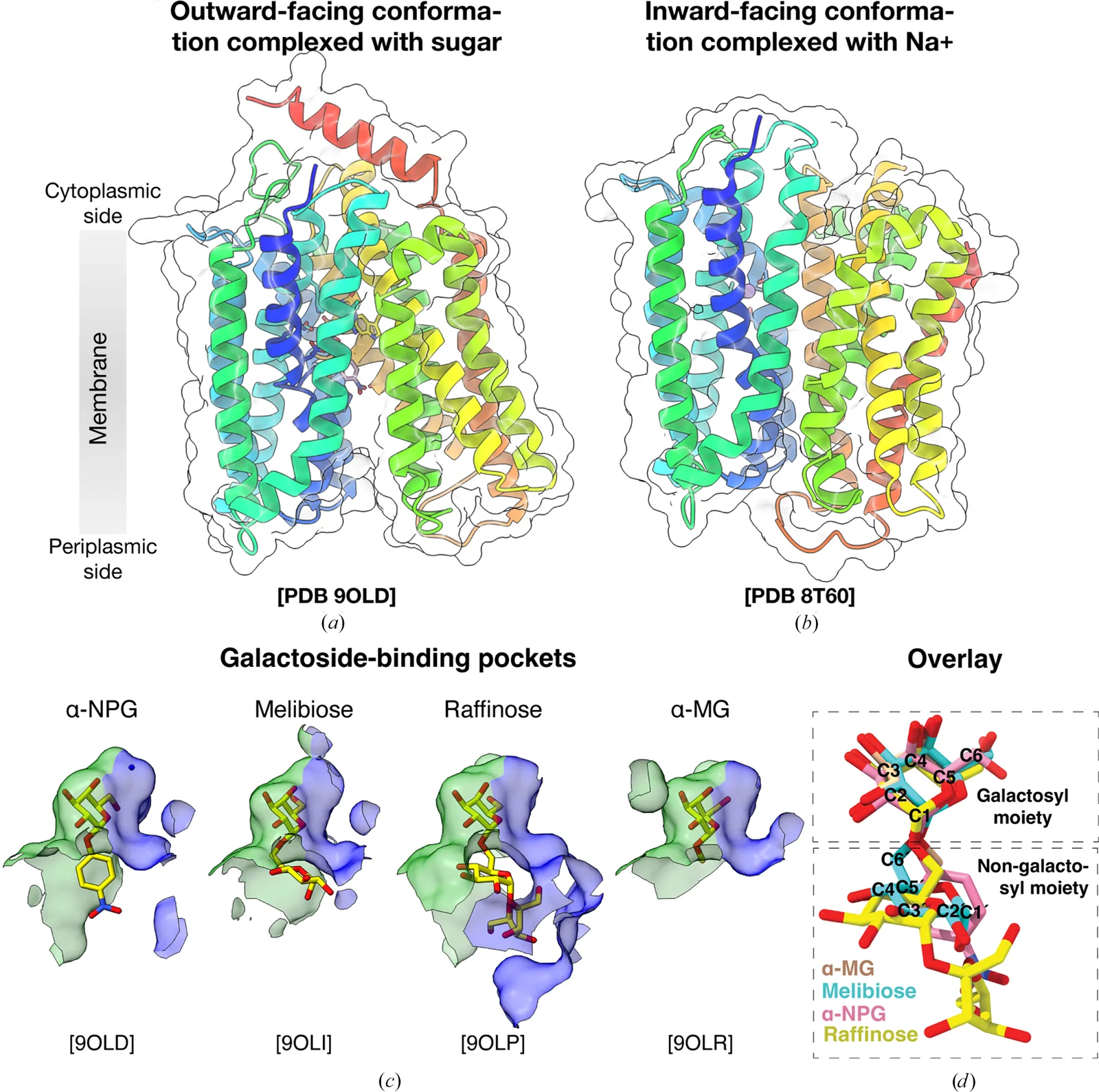
Structures of MelB_St_. (*a*) Crystal structure of the sugar-bound outward-facing conformation of the MelB_St_ D59C uniporter mutant at a resolution of 2.60 Å (PDB entry 9old). The bound α-nitrophenyl galactoside (α-NPG, pink) interacts with MelB_St_ at the apex of the outward-facing cavity and in the middle of the protein. The α-NPG-binding residues are highlighted as sticks. (*b*) CryoEM structure of the Na^+^-bound inward-facing conformation of WT MelB_St_ (PDB entry 8t60). The structure was solved as a complex with a conformation-specific nanobody Nb725_4 and a fiducial NabFab. The bound Na^+^ is shown as a pink sphere and the binding residues are highlighted as sticks. The membrane sidedness is indicated. (*c*) Sugar recognition pocket. Crystal structures of four α-sugars with different numbers of sugar units. All sugars are colored yellow, and the binding pocket contributed by the N- and C-terminal domains is colored green and blue, respectively. (*d*) Four well aligned bound sugar molecules. The four crystal structures shown in (*a*) were overlaid and the bound sugar structures are displayed with identical color codes. The carbon positions for the galactosyl and glucosyl moieties of melibiose are labeled C1–C6 and C1′–C6′, respectively. This panel was modified from Figs. 2 and 4(*e*) of Hariharan *et al.* (2026 ▸)

X-ray crystallography and cryoEM single-particle analysis revealed that this transporter adopts the typical fold of most MFS transporters, with 12 transmembrane helices forming two domains of six helices each, surrounding a hydrophilic cavity. The central cavity is accessible from only one side of the membrane in either the outward- or inward-facing conformation (Figs. 1 ▸*a* and 1 ▸*b*). By cycling between inward- and outward-facing states via a ‘rocker-switch’ mechanism, the substrate can cross the transport protein through the discontinuous path formed in either the outward- or inward-facing conformation.

X-ray crystallography has revealed the outward-facing apo or substrate-bound state (Guan & Hariharan, 2021 ▸; Hariharan, Bakhtiiari *et al.*, 2024 ▸; Hariharan *et al.*, 2026 ▸), which is suggested to be the lowest free-energy state (Guan & Hariharan, 2021 ▸; Hariharan, Shi *et al.*, 2024 ▸; Hariharan *et al.*, 2026 ▸) and has recently been confirmed by the simulated minimum free-energy landscape of melibiose translocation in MelB_St_ (Liang & Guan, 2024 ▸). The thermostable mutant D59C, with a single-site mutation at the cation-binding site, is a valuable tool for crystallization to determine the sugar-specificity determinants of the pocket, as discussed further in the following section. The cryoEM structure was obtained using wild-type (WT) MelB_St_, which reveals the inward-facing conformation in an Na^+^-bound state (Hariharan, Shi *et al.*, 2024 ▸), and this high-energy conformation was stabilized by the conformation-specific binder nanobody-725 (Nb725_4; Katsube *et al.*, 2023 ▸; Bloch *et al.*, 2021 ▸). HDX-MS analysis indicated that this nanobody can completely inhibit the dynamics of most transmembrane regions of MelB_St_ bound with Na^+^ for at least 50 min (Hariharan, Shi *et al.*, 2024 ▸), and functional studies also showed that it completely inhibited the melibiose transport activity mediated by MelB_St_ when co-expressed in *Escherichia coli* cells (Katsube *et al.*, 2023 ▸). Nb725 binding shifts conformations toward the inward-facing state, making it a useful tool for structural and functional studies that have provided critical insights into the transport mechanisms, as discussed in later sections.

### 2.1. Thermostable mutants of D55C or D59C MelB_St_

Most crystal structures with substrate bound were determined using the D59C mutant (Guan & Hariharan, 2021 ▸; Hariharan, Bakhtiiari *et al.*, 2024 ▸; Hariharan *et al.*, 2026 ▸). Mutations at the cation-binding site Asp55 or Asp59, replacing Asp with Cys or Ala, significantly alter MelB_St_ stability and function (Hariharan & Guan, 2021 ▸). Purified D55C or D59C MelB_St_ in undecyl-β-d-maltopyranoside (UDM) detergent solutions exhibited greatly improved thermostability. The melting temperature (*T*_m_) of the D55C or D59C mutants, determined by thermal denaturation using circular-dichroism (CD) spectroscopy, was ∼56.75°C or ∼60°C (Guan & Hariharan, 2021 ▸), which is 2°C or 6°C higher than for WT MelB_St_ in the presence of Na^+^. Higher quality crystals could be easily produced from the thermostable mutants, making them valuable tools for investigating sugar recognition by MelB_St_ (Guan & Hariharan, 2021 ▸; Hariharan, Bakhtiiari *et al.*, 2024 ▸; Hariharan *et al.*, 2026 ▸).

Both thermostable mutants significantly alter MelB_St_ function in distinct ways. The D59C mutant is a uniporter (Guan & Hariharan, 2021 ▸). It mediates the downhill translocation of melibiose (Guan & Hariharan, 2021 ▸; Ethayathulla *et al.*, 2014 ▸), the native substrate, and α-nitrophenyl galactoside (α-NPG; Guan & Hariharan, 2021 ▸; Hariharan *et al.*, 2026 ▸), a sugar analog. Thus, the D59C mutant can move the sugar substrate based on its own concentration gradient, but it is unable to catalyze active melibiose transport against its concentration gradient, which requires the coupling of either H^+^, Na^+^ or Li^+^, since there is no detectable binding for Na^+^ and Li^+^. The D55C mutant lost melibiose transport when coupled to Na^+^ and Li^+^ but exhibited slightly increased H^+^-coupled melibiose accumulation, behaving as an H^+^-coupled transporter (Hariharan, Bakhtiiari *et al.*, 2024 ▸). The features in coupling and functional changes of the two mutants indicate that the cation-binding pocket is an inserted module that influences existing sugar binding and translocation and modifies transport kinetics.

## 3. Sugar recognition and affinity regulation

### 3.1. Binding specificity

Binding assays showed that MelB_St_ recognizes disaccharides or trisaccharides containing a galactosyl moiety, and the native substrate, which functionally co-evolved with MelB, is melibiose, a disaccharide formed from galactose and glucose linked by an α-1,6-galactosyl bond [d-Gal-(α1→6)-d-Glc] (Hariharan *et al.*, 2026 ▸; Guan *et al.*, 2011 ▸; Hariharan & Guan, 2014 ▸). The sugar analog α-NPG binds MelB_St_ with much higher affinity and serves as a useful substrate for studies of the binding site. We have resolved several crystal structures of the D59C mutant complexed with α-galactosides with varied sugar units, including methyl α-d-galactoside with a single unit, melibiose with two units, raffinose with three units and α-NPG (Hariharan *et al.*, 2026 ▸). The crystal structures of both D55C and D59C MelB_St_ complexed with the melibiose-based detergent dodecyl-β-melibioside (DDMB) were also determined (Guan & Hariharan, 2021 ▸; Hariharan, Bakhtiiari *et al.*, 2024 ▸). All of these structures adopt a virtually identical outward-facing conformation, and the sugar molecules from the overlaid structures show that the bound galactosyl moieties are also aligned well in the specificity-determinant pocket, regardless of the number of monosaccharide units (Figs. 1 ▸*c* and 1 ▸*d*). The structural evidence firmly supports the conclusion that the galactosyl moiety determines the specificity of the primary substrates and that the nongalactosyl moiety contributes to the binding affinity.

### 3.2. Molecular interactions of galactoside binding

**Figure 2:**
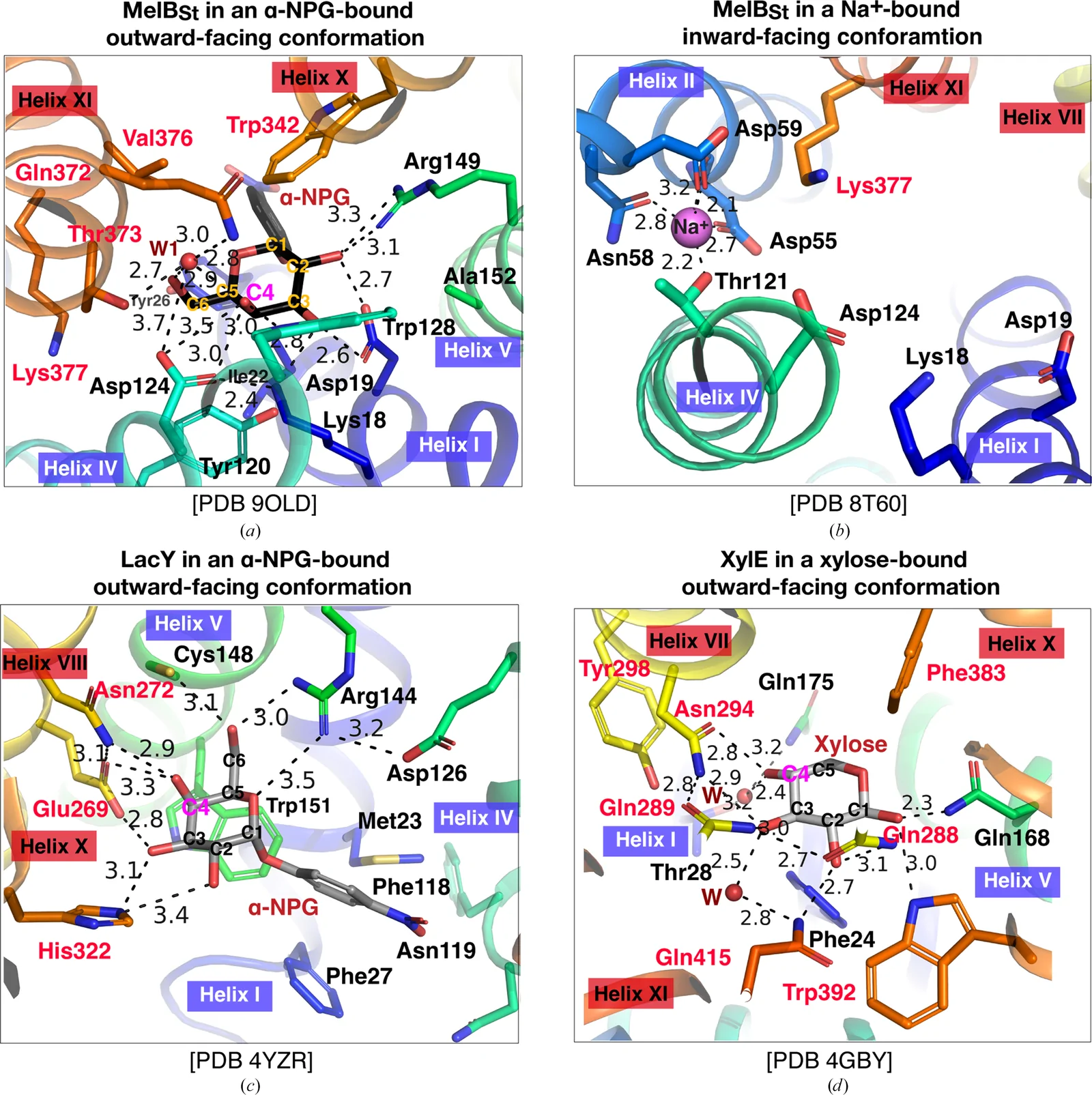
Sugar recognition of MelB_St_, LacY and XylE, and cation recognition of MelB_St_. In all panels, the N- and C-terminal helices are labeled on a blue or red background, respectively. The residues from the N- and C-terminal helices are labeled in black and red, respectively. (*a*) Galactoside recognition in MelB_St_. The α-NPG-bound outward-facing structure at the currently highest resolution (PDB entry 9old) is used to illustrate galactoside binding. C1–C6 on the galactopyranosyl ring are labeled. Wat 1, water molecule in the sugar recognition site. (*b*) Na^+^ recognition in MelB_St_. The Na^+^-binding pocket is shown from the inward-facing structure of WT MelB_St_ (PDB entry 8t60). The bound Na^+^ is shown as a red sphere. (*c*) Galactoside recognition in LacY. The figure was generated using the outward-facing α-NPG-bound conformation (PDB entry 4zyr). C1–C6 on the galactopyranosyl ring of α-NPG are labeled. (*d*) Xylose recognition in XylE. The figure was generated using the outward-facing xylose-bound conformation (PDB entry 4gby). C1–C5 on the xylofuranosyl ring are labeled. W, water molecule shown as a red sphere.

The binding pocket comprises 14 residues distributed across five helices in both the N- and C-terminal domains (Guan & Hariharan, 2021 ▸; Hariharan *et al.*, 2026 ▸; Fig. 2 ▸*a*). The N-terminal residues, comprising Lys18, Asp19, Ile22 and Tyr26 (helix I), Tyr120, Asp124 and Trp128 (helix IV), and Arg149 and Ala152 (helix V), contribute to binding the galactosyl moiety. The charged residues Asp19, Asp124 and Arg149 form multiple hydrogen bonds with the hydroxyl groups on C2, C3, C4 and C6 of the galactopyranosyl ring. The C-terminal residues, including Trp342 (helix X) and Gln372, Thr373, Val376 and Lys377 (helix XI), primarily shape the binding pocket, with limited direct interaction with the sugar molecules, except for Trp342. The water molecule (Wat 1) bound to the C4-OH and C6-OH groups of the galactopyranosyl ring forms hydrogen bonds to the C-terminal residues Thr373 and Gln372 (helix XI) and the N-terminal Asp124 (helix IV), thereby stabilizing the binding of C4-OH to Asp124 and Trp128. Notably, MelB does not recognize glucose or glucosides, and binding of the C4-OH group is crucial for distinguishing galactose from glucose.

The C3-OH and C2-OH groups on the galactopyranosyl ring form multiple hydrogen-bonding interactions with the charged residues Lys18, Asp19 and Arg149 in helices I and V, which play a crucial role in stabilizing the recognition of OH-4 and enhancing the binding affinity.

### 3.3. Arg149 plays a dual role in binding and gating

**Figure 3:**
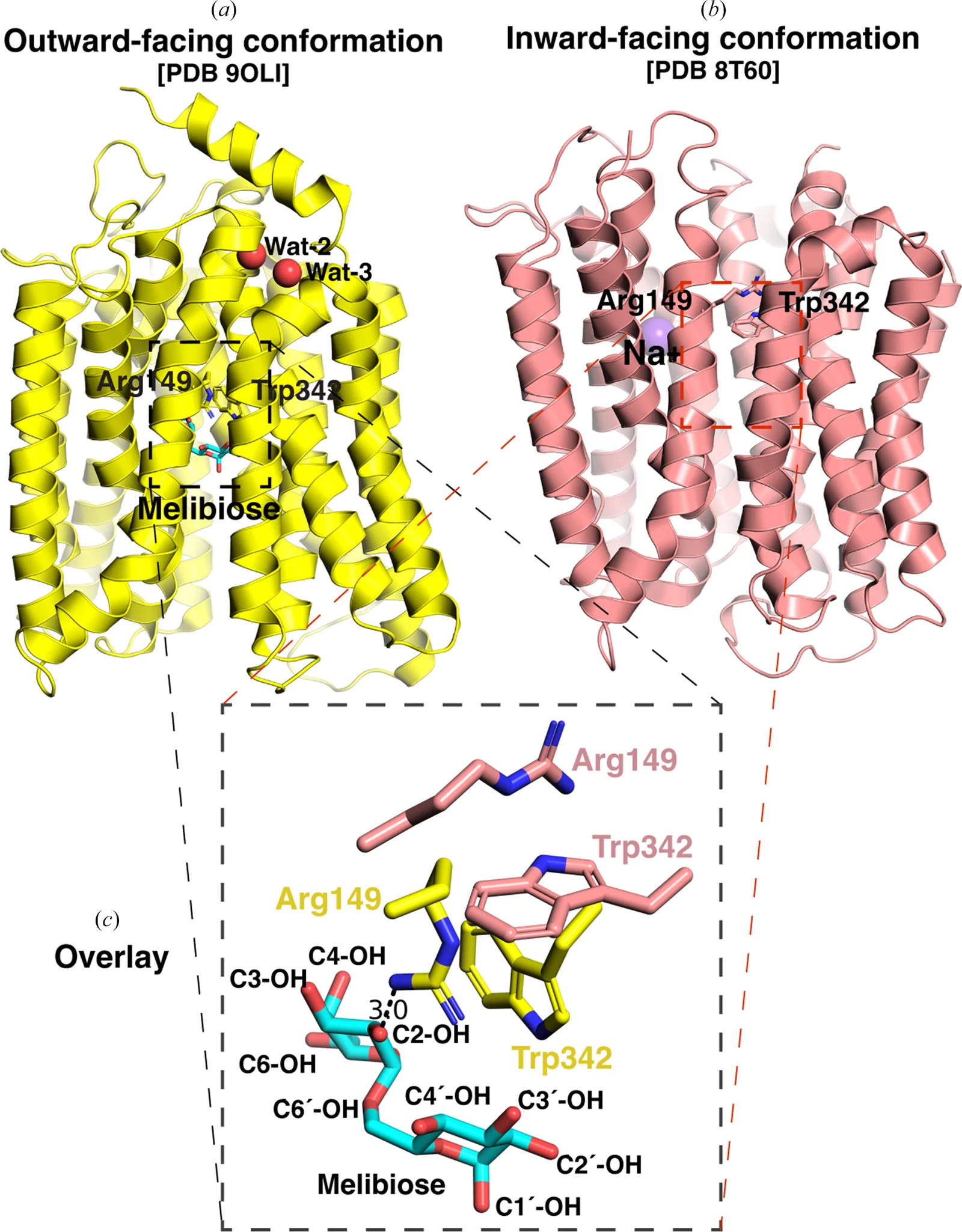
Arg149 is a binding residue and also a gating residue. (*a*) Bent Arg149 at the sugar-binding state. A bent Arg149 is illustrated from the melibiose-bound outward-facing conformation (PDB entry 9olp). Water molecules (Wat) are colored red. (*b*) Extended Arg149 in the sugar low-affinity inward-facing conformation (PDB entry 8t60). Na^+^ is shown as a blue sphere. (*c*) Overlay. The boxed region in (*a*) and (*b*) was enlarged as indicated. Arg149 and Trp342 from the higher affinity sugar-bound and empty low-affinity conformations are colored yellow and pink, respectively; the melibiose molecule bound to the outward-facing structure is colored cyan.

Arg149, located at the edge of this sugar-binding pocket, is also a gating side chain on helix V (Fig. 3 ▸*b*). In the outward-facing conformation, this side chain is restrained by a cation–π interaction with Trp342 on the C-terminal helix X, another binding residue in this binding pocket, forcing the Arg149 side chain into a bent conformation regardless of the absence (PDB entry 8frh) or presence (PDB entries 7l16, 9oli and 9old) of sugar. This bent configuration is the binding configuration. In the inward-facing conformation (PDB entry 8t60), both Trp342 and Arg149 are displaced due to the separation of helices V and X at the cytoplasmic side. The Arg149 side chain at the open cytoplasmic gate gains space and conformational freedom, adopting an extended conformation and moving away from the sugar-binding pocket, thereby contributing to reduced binding affinity in the inward-facing state. The importance of these side-chain structural rearrangements for sugar affinity will be discussed further.

### 3.4. Mutational analysis of the sugar-binding pocket

Cysteine-scanning mutagenesis was conducted by placing a Cys residue at each position one at a time in a functional Cys-less MelB_St_ mutant with all eight endogenous Cys residues replaced by Ala (Markham *et al.*, 2021 ▸). The transport assays showed that all positions in the sugar-binding pocket are sensitive to Cys mutation, except Ala152, Thr373 and Val376, which do not directly contact the sugar molecule. Single-Cys mutants at five charged positions (Lys18, Asp19, Asp124, Arg149 and Lys377) failed in melibiose active transport, and also showed poor melibiose downhill transport, except for the R149C mutant, which showed weak fermentation activity. The single-site mutants at those positions also showed poor transport activities, except for Lys377, which showed partial inhibition (Ethayathulla *et al.*, 2014 ▸).

### 3.5. Conformational changes influence sugar binding but not Na^+^ binding

Label-free binding-assay isothermal titration calorimetry (ITC) measurements have been extensively used to determine the binding of melibiose to MelB_St_ (Ethayathulla *et al.*, 2014 ▸; Hariharan & Guan, 2014 ▸) and the coupling cations Na^+^ and Li^+^ (Hariharan & Guan, 2017 ▸; Guan & Hariharan, 2021 ▸). The proton affinity in the absence or presence of melibiose was also analyzed by fitting the apparent dissociation constants (*K*_d_) of Na^+^ as a function of H^+^ concentration (Hariharan & Guan, 2017 ▸). p*K*_a_ values of 6.25 and 6.59 in the absence and presence of melibiose, respectively, were obtained. These p*K*_a_ values fall within the slightly acidic range, indicating that only a small fraction of MelB_St_ is protonated at the physiological pH of approximately 7.6, and MelB_St_ functions based on H^+^ mode are not optimal. Furthermore, melibiose affinity is poor in the apo state, but is significantly increased in the presence of Na^+^ or Li^+^ by eightfold or fourfold, respectively, and the Na^+^ or Li^+^ affinity is also increased by melibiose with the same magnitudes, which was defined as positive cooperativity (Hariharan & Guan, 2017 ▸; Guan & Hariharan, 2021 ▸).

The structural dynamics and conformational changes of membrane transporters are essential for their transport functions, and transport proteins undergo varying degrees of structural motions and conformational equilibration among different states. The affinity for a specific conformation cannot be determined from binding assays without isolating the conformational state. Thus, the measured binding is likely to be an average across all conformations. A conformation-selective nanobody is very useful in this regard. Using the conformation-specific nanobody (Nb725 or Nb725_4), we isolated the inward-facing conformation of MelB_St_ for cryoEM structural and biophysical analyses (Katsube *et al.*, 2023 ▸; Hariharan, Shi *et al.*, 2024 ▸). Nb725_4 is a mutant of Nb725 with a different scaffold sequence, engineered to interact with an Nb-binding Fab (NabFab) via grafting (Bloch *et al.*, 2021 ▸); *i.e.* both Nb725_4 and Nb725 bind the same site on MelB_St_ and Nb725_4 can bind the NabFab (Hariharan, Shi *et al.*, 2024 ▸). Our data showed that Nb725_4-bound MelB_St_ in the inward-facing conformation does not bind melibiose well. While melibiose binding was undetectable by ITC, the Na^+^ binding was only marginally reduced. Testing the higher affinity α-NPG revealed a 30-fold decrease in sugar binding compared with an Nb-free MelB_St_. *In vivo* co-expression of this Nb and MelB_St_ showed no detectable melibiose uptake (Katsube *et al.*, 2023 ▸) and HDX-MS analysis showed complete inhibition against deuterium uptake for at least 50 min (Hariharan, Shi *et al.*, 2024 ▸). The data support the conclusion that the sugar-binding affinity of MelB_St_ is influenced by conformational change, in addition to cation binding, and that Na^+^ binding is affected only by sugar binding.

Structurally, comparing the N-terminal domain between the inward- and outward-facing conformations shows that the galactosyl recognition pocket is broken due to the separation of the binding helices I, IV and V in the N-terminal domain from helices X and XI in the C-terminal domain. Along with this domain-separation processing (Hariharan, Shi *et al.*, 2024 ▸), the side chains at several positions, particularly Arg149 and Gln372, exhibit greater displacement. Their concerted motions of sugar-binding side chains are coupled to the domain alterations as part of a larger-scale conformational transition (Fig. 3 ▸). As described above, in the outward-facing conformation the bent Arg149 interacts with the C2-OH group of the galactosyl moiety; in the inward-open conformation Arg149 is significantly displaced away from the C2-OH group. These structural changes are consistent with the measured affinity when trapping MelB_St_ in the inward-facing state, supporting the notion that sugar binding is regulated by conformational dynamics and transition (Hariharan, Shi *et al.*, 2024 ▸).

### 3.6. Comparison with the H^+^-coupled lactose permease LacY

The *E. coli* lactose permease (LacY; Guan & Kaback, 2006 ▸; Guan, 2023 ▸), a well studied H^+^-coupled MFS symporter, was examined for comparison. This H^+^-coupled galactoside symporter exhibits substrate specificity similar to MelB_St_, and both can transport α- and β-galactosides but not glucosides (Guan & Kaback, 2006 ▸). In both cases, the galactosyl moiety determines specificity, and different adducts at the anomeric carbon C1 of the galactopyranosyl ring increase binding affinity. For example, α-NPG, which has an aromatic phenyl group, shows much higher affinity for both transporters than their natural substrates lactose or melibiose (Sahin-Tóth *et al.*, 2001 ▸; Hariharan *et al.*, 2026 ▸). Although glucose is an epimer of galactose, differing only at C4, neither glucose nor glucosides are recognized by LacY or MelB.

The crystal structure of the outward-facing conformation of a LacY mutant with α-NPG bound has revealed a well defined galactoside-binding pocket situated between the N- and C-terminal domains (Fig. 2 ▸*c*; Kumar *et al.*, 2015 ▸). Similar to MelB, five helices are involved in the binding; in LacY, three of these are from the N-terminal helices (I, IV and V) and two are from the C-terminal helices (VIII and X). The binding sites are composed of charged, polar and aromatic residues, with each OH group at the C2, C3, C4 or C6 positions forming at least one hydrogen bond to a charged residue. The chemical composition of this galactoside-binding pocket in LacY is comparable to that in MelB_St_. Interestingly, in MelB_St_ the specificity-determining group C4-OH of the galactosyl ring is hosted by the N-terminal helices IV and XI; in LacY, C4-OH interacts with the C-terminal helix VIII. The α-NPG molecule in both transporters is positioned at a similar level relative to the lipid bilayer but is oriented differently within their respective cavities. Notably, the C4-OH groups are hosted by helices in the domain that also host their cation-binding site. This structural arrangement may secure the coupling between the primary substrate and the co-substrate.

### 3.7. Comparison with the H^+^-coupled xylose permease XylE

Xylose is a five-carbon sugar, and its C2-OH, C3-OH and C4-OH configurations are structurally identical to those in glucose. The *E. coli* H^+^- coupled xylose permease XlyE is also an MFS transporter with 12 transmembrane helices. The crystal structure of the outward-facing conformation of xylose-bound XlyE also showed that five helices are involved in sugar binding (Sun *et al.*, 2012 ▸). The N-terminal helices I and V and the C-terminal helices VII, X and XI host the binding residues. Interestingly, helices I, V and X are involved in sugar binding in all three of the bacterial sugar permeases MelB, LacY and XlyE. A major difference is the lack of charged residues in the XlyE sugar-binding pocket. All OH groups form hydrogen-bonding interactions with polar residues, especially Gln. Two water molecules are identified. Interestingly, the water bound to C4-OH is near the proposed protonation site on helix I. This feature is similar to that observed in MelB_St_ (Hariharan *et al.*, 2026 ▸), where a water molecule is part of recognition and is located between C4-OH/C6-OH of the galactoside and the cation site.

## 4. Cation selectivity and recognition of MelB_St_

Compared with the large sugar-binding pocket formed by both transmembrane domains, the cation-binding pocket is hosted by only two helices (II and IV) within the N-terminal domain (Fig. 2 ▸*b*). This cation-binding pocket binds H^+^ and the alkali metals Na^+^ or Li^+^, but does not recognize other alkali metals including potassium ion (K^+^), rubidium ion (Rb^+^) and caesium ion (Cs^+^) (Guan *et al.*, 2011 ▸). Extensive biophysical analyses and structural studies support the conclusion that the cation-binding pocket, formed by two negatively charged side chains of Asp55 and Asp59 and two polar side chains of Asn58 and Thr121, hosts Na^+^, H^+^ or Li^+^ (Hariharan & Guan, 2017 ▸; Guan & Hariharan, 2021 ▸; Katsube *et al.*, 2022 ▸; Hariharan, Bakhtiiari *et al.*, 2024 ▸). In this shared cation-binding pocket, Asp59 serves as the binding site for any of the three cations, while Asp55 binds only Na^+^ or Li^+^, and Thr121 is required only for Na^+^ binding, not for Li^+^. Lys377 sits between the sugar- and cation-binding pockets. In the inward-facing conformation Lys377 interacts with Asp59, and in the outward-facing conformation of the D59C mutant Lys377 interacts with Asp55. Notably, there is no direct contact between the cation-binding site and the sugar-binding site, but the sugar-binding residue Asp124 is 7–8 Å from Asp55 and Asp59. The observed effect of Na^+^ on sugar-binding affinity has been identified through allosteric effects, as discussed below.

## 5. Functionally important gating salt-bridge network stabilizing the inner barrier

**Figure 4:**
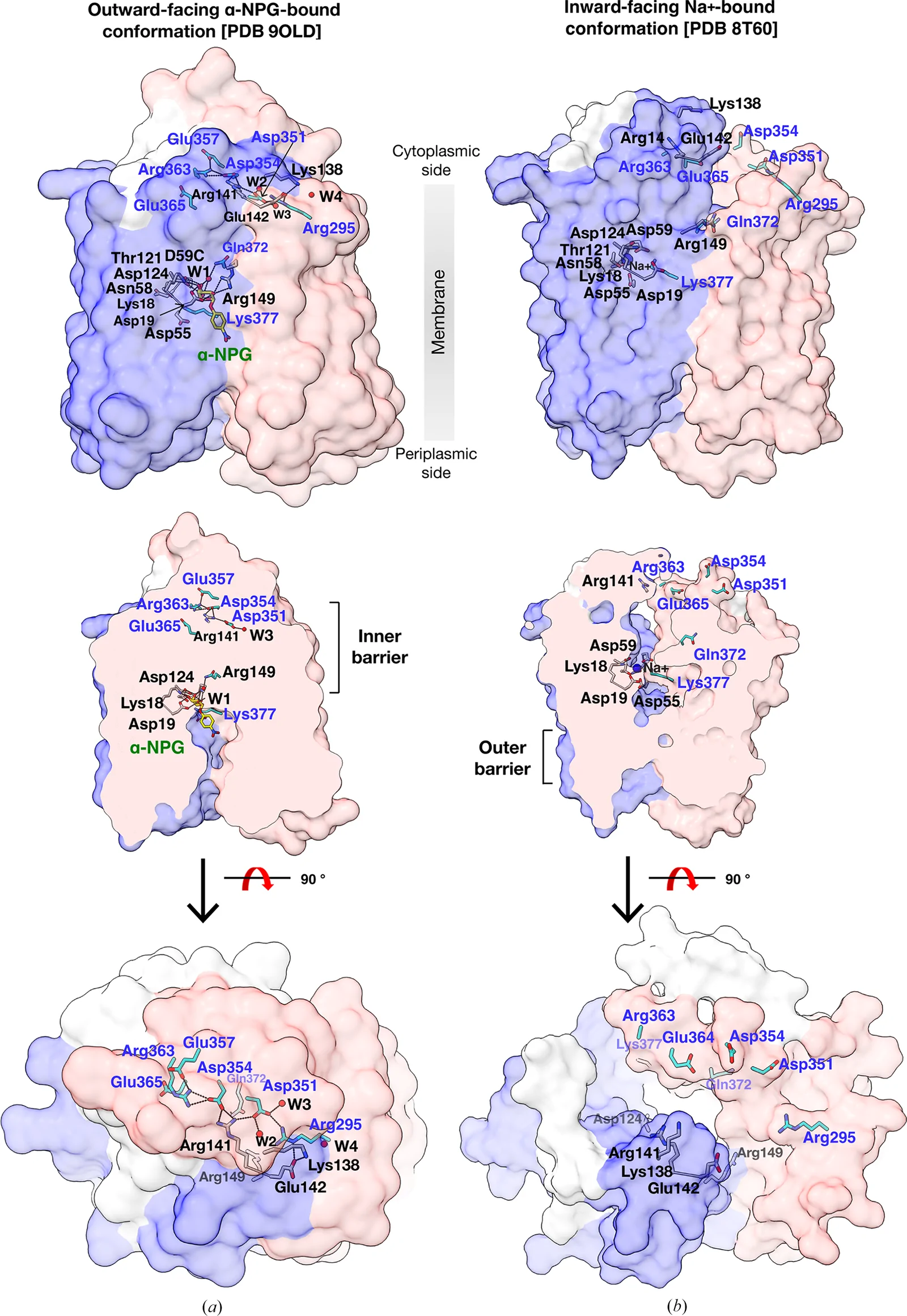
Cytoplasmic gating salt-bridge network. (*a*) Outward-facing α-NPG-bound conformation (PDB entry 9olp). The α-NPG molecule is colored yellow. (*b*) Inward-facing Na^+^-bound conformation (PDB entry 8t60). Na^+^ is colored as a blue sphere. Upper row: side view with the cytoplasmic side at the top. The membrane sidedness is labeled. Middle row: a cross-section of the surface representations of both structures from the upper row. The substrate-translocation inner barrier in the outward-facing structure and the outer barrier in the inward-facing structure are indicated, respectively. Lower row: cytoplasmic view. The residues in both the sugar- and cation-binding pockets in the middle of the protein and the cytoplasmic gating salt-bridge network from the N- and C-terminal domains are colored gray and cyan, respectively, and labeled in black and blue, respectively. Dashed lines indicate either interaction between sugar and protein or salt-bridge interactions.

Integration of the outward- and inward-facing structures and site-directed mutagenesis showed that the salt-bridge network seals the cytoplasmic gate formed between the N- and C-terminal domains and stabilizes the inner barrier (Guan & Hariharan, 2021 ▸; Fig. 4 ▸*a*), and plays a critical role in transport kinetics (Amin *et al.*, 2014 ▸). The two negatively charged residues, Asp351 and Asp354, in the C-terminal helix X are functionally critical for melibiose transport (Markham *et al.*, 2021 ▸). Structurally, they form salt-bridge interactions with the N-terminal Arg141 in helix IV and with the C-terminal Arg295 and Arg363 in helix IX and loop_10–11_, respectively. Arg295 also forms multiple contacts with Lys138 and Glu142, in addition to Asp351. The high-resolution crystal structure revealed that three water molecules are engaged in this network (Hariharan *et al.*, 2026 ▸). Notably, Arg149 is located within this gate region including positions 138–142. In the inward-facing conformation these interactions between the two domains are broken (Fig. 4 ▸*b*).

## 6. Conformational dynamics and influence of substrate binding

The robust HDX-MS can map the ordered and disordered regions of a full-length protein, as well as ligand-induced structural dynamics and conformational changes. This method was used to analyze the conformational dynamics of MelB_St_ and the effects of melibiose binding in the absence or presence of Na^+^, Na^+^ alone or Nb725 (Hariharan, Shi *et al.*, 2024 ▸; Hariharan *et al.*, 2026 ▸). Na^+^-bound MelB_St_ favors an outward-facing conformation, whereas the Nb725-trapped MelB_St_ is in an inward-facing conformation. A differential study in the absence or presence of Nb725 was used to explore the conformational transition between the two states. Under all conditions, both the N- and C-terminal tails exhibited high HDX rates, particularly in disordered regions that lack structural characterization by crystallography or cryo-EM (Guan & Hariharan, 2021 ▸; Hariharan, Shi *et al.*, 2024 ▸).

**Figure 5:**
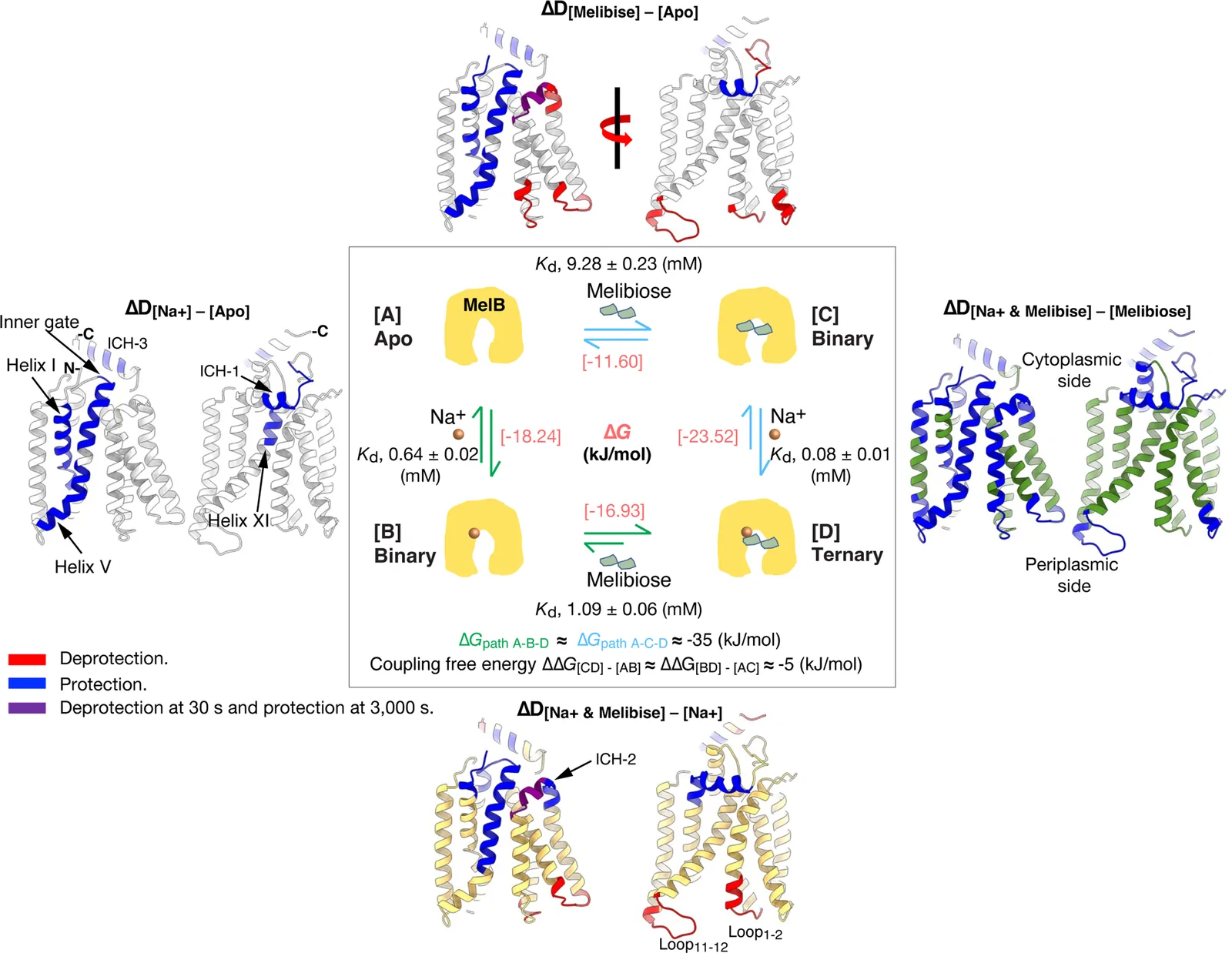
Differential deuteriation in the thermodynamic cycle. The determined binding *K*_d_ values (m*M*) of melibiose or Na^+^ in the absence or presence of the other are presented in the binding thermodynamic cycle scheme, adapted from Fig. 6 of Hariharan & Guan (2017 ▸). The differential deuteriation was calculated from the deuterium uptake (D) at 30, 300 and 3000 s in two states as indicated. All data (>threshold and *P* < 0.05) were mapped to the outward-facing structure and displayed in two orientations related by 180°, as indicated. N- and C-termini are labeled, and membrane orientation is indicated. Blue-colored regions, protection; red-colored regions, deprotection; purple-colored region, deprotection at 30 s and protection at 3000 s.

Differential deuterations for the effects induced by melibiose and/or Na^+^ were analyzed and mapped to the structures, which are presented on the thermodynamic cycle scheme for cooperative binding of melibiose and Na^+^ (Fig. 5 ▸; Hariharan *et al.*, 2026 ▸). Na^+^ binding to the apo state primarily provided HDX protection, including protection of the sugar-binding helices I and V and the positions at the gating salt-bridge network shown in blue. For Na^+^ binding to the melibiose-bound binary complex, inhibitory effects were found in more areas. Binding of melibiose to the apo state caused HDX protection in regions similar to those protected by Na^+^, but also induced deprotection in peripheral loops, as shown in red.

Interestingly, binding of melibiose to the binary complex of MelB/Na^+^, in addition to enhancing the inhibitory effect on conformational flexibility of the Na^+^-inhibited regions, such as helices I and V and ICH-1 in the middle loop, also increases the conformational dynamics of several loops, including the loops located at the interface of the N- and C-terminal domains, such as loop_1–2_ and loop_11–12_. The purple-colored region shows that earlier deprotection and longer incubation led to protection. These deprotection regions were associated only with melibiose binding, suggesting conformational dynamics and changes induced by the primary substrate melibiose. The detailed integration of HDX and conformation is described below.

### 6.1. Structural dynamics in the apo state

**Figure 6:**
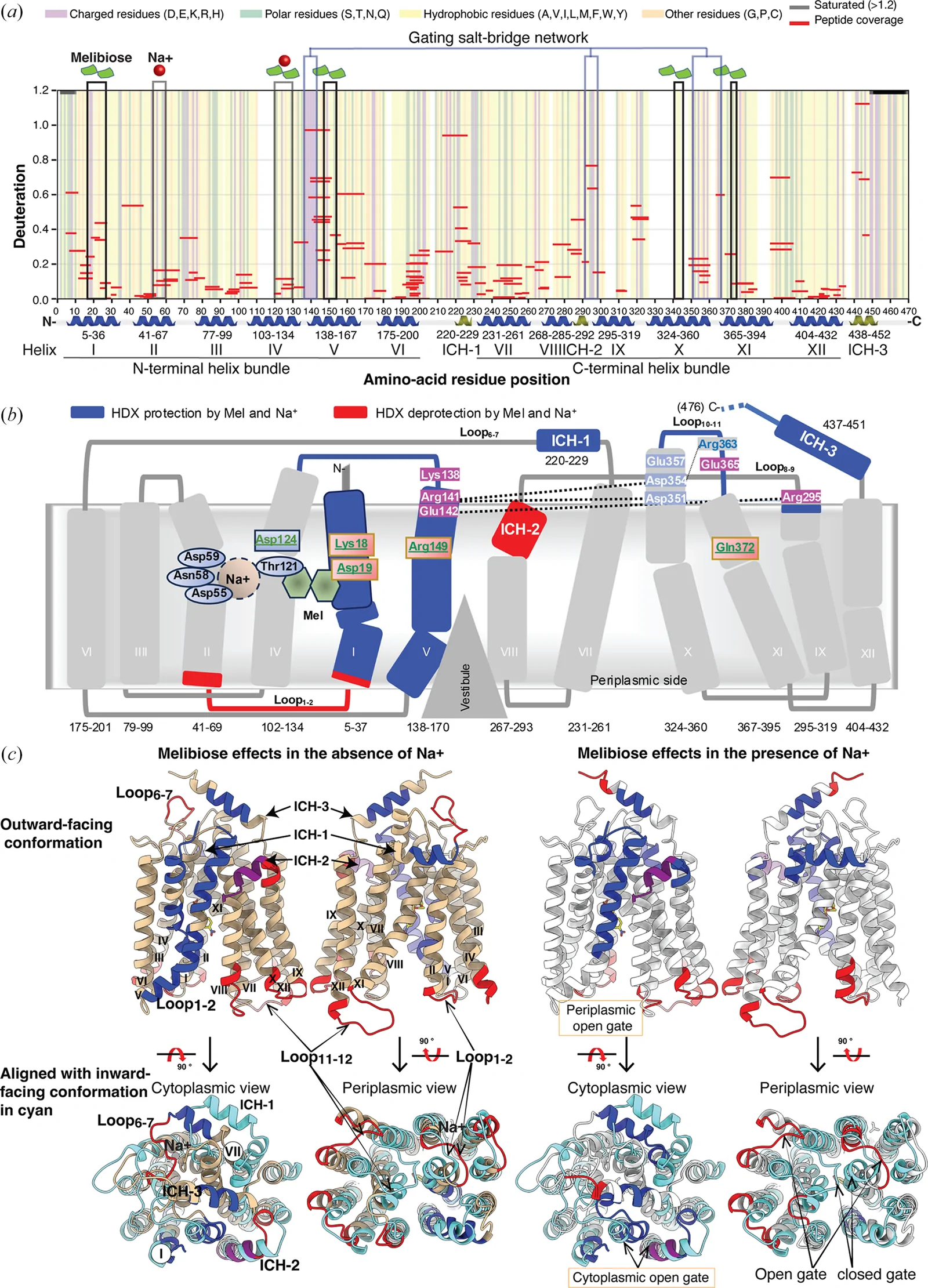
Mapping of HDX of MelB_St_ in the absence or presence of melibiose and/or Na^+^. (*a*) Deuterium map of apo MelB_St_. Mean deuteriation levels of MelB_St_ peptides in the apo state are presented against the amino-acid sequence. The lengths of the red bars correspond to the peptide lengths, and the white background indicates noncovered positions. Peptides covering sugar- and cation-binding sites and the cytoplasmic gating salt-bridge network are highlighted by boxes and labeled individually. (*b*) Mapping of ligand effects of HDX on a topology model of MelB_St_. Melibiose- and Na^+^-binding residues are labeled in green or black, respectively, and residues in the cytoplasmic gating salt-bridge network are labeled in white. Residues in pink at the sugar-binding pocket or on magenta backgrounds in the cytoplasmic gating salt-bridge network indicate higher deuteriation levels in the apo state with significant ligand-induced protection. Residues on a blue background indicate peptides with low levels of deuteriation in the apo state with no significant ligand effects. Arg363 is a noncoverage position. (*a*) and (*b*) were modified from Figs. 5(*a*) and 8(*c*), respectively, of Hariharan *et al.* (2026 ▸). (*c*) Melibiose-induced differential HDX mapping on structures of MelB_St_. Left and right columns, melibiose effects on HDX of MelB_St_ in the absence or presence of Na^+^, respectively. Upper rows, a side view of the α-NPG-bound outward-facing structure (PDB entry 9olp) with helix V or II at the front, respectively, as labeled. Lower row, the same structure was aligned with the inward-facing structure (PDB entry 8t60) in cyan and rotated 90° around the membrane plane. Overlapping peptides in red or blue indicate deprotection or protection, respectively. Loops are colored red. The purple-colored peptide indicates deprotection at the 30 s time point and protection at the 3000 s time point. The displacement of loop_1–2_ and loop_11–12_ between the inward- and outward-facing conformations is indicated by arrows.

Deuterium uptake in the apo state revealed the conformationally flexible regions of MelB_St_. Six regions with higher deuterium uptake were identified; three encompass sugar-binding residues and the others are distributed in the loops of the transmembrane domain (Fig. 6 ▸*a*). Interestingly, peptides covering the Na^+^-binding residues and the sugar-binding residues near the Na^+^-binding pocket show low deuteration, indicating limited solvent accessibility in those regions critical for cation and substrate recognition (Hariharan *et al.*, 2026 ▸).

### 6.2. Different effects of substrate binding at the sugar- and cation-binding sites

Deuteriation influenced by the binding of melibiose, Na^+^ or both showed similar profiles, indicating that the dynamic changes occurred in the same regions (Hariharan *et al.*, 2026 ▸). The extent and magnitude of these changes varied, with minor effects from melibiose alone, more significant effects from Na^+^ alone and the strongest effects when both were present. Peptides that cover the cation-binding residues and residues in the sugar-binding site with low HDX rates in the apo state did not show significant changes in the presence of either melibiose, Na^+^ or both, indicating conformational rigidity; on the other hand, peptides covering the sugar-binding residues with higher HDX rates were sensitive to the binding of either substrate (Figs. 2 ▸, 6 ▸*b* and 6 ▸*c*). In general, inhibition by melibiose was the weakest, that by Na^+^ was greater than that by melibiose and that of melibiose combined with Na^+^ was stronger than each individually, consistent with cooperativity.

### 6.3. Substrate-induced dynamic changes in the cytoplasmic salt-bridge network

The effects on the cytoplasmic salt-bridge network can be categorized into two groups: (i) the less flexible positions in the apo state, including three negatively charged residues at positions Asp351, Asp354 and Glu357 on helix X, exhibited little change in the presence of melibiose with or without Na^+^ or Na^+^ alone, while (ii) the flexible positions, including Arg141, Lys138, Glu142, Arg295 and Glu365, showed varying degrees of inhibition by melibiose or Na^+^, especially in the presence of both (Figs. 4 ▸ and 6 ▸*b*). Unfortunately, Arg363 was not covered in this HDX-MS study because no labeled peptide spanned this position.

The allosteric inhibition of conformational flexibility in these gating regions likely reflects stronger interactions between the N- and C-terminal charged residues, indicating that the inner barrier is stabilized by the substrates. Thus, binding of the substrate(s) favors the outward-facing conformation (Guan & Hariharan, 2021 ▸; Liang & Guan, 2024 ▸; Hariharan *et al.*, 2026 ▸). Data for inhibition of the cytoplasmic gating region were missing from the Nb-free versus Nb-bound differential HDX-MS study (Hariharan, Shi *et al.*, 2024 ▸) because the N-terminal charged residues in this charged network are also the binding site for Nb725_4, so the observed inhibition could result from the nanobody binding per se. Overall, out studies support the conclusion that binding of the primary substrate or co-substrate, especially when both bound, shifts the conformational equilibrium of MelB_St_ toward the outward-facing conformation. On the other hand, the inhibition of conformational dynamics affects only primary substrate binding, with no significant effect on the coupling cation binding.

### 6.4. Increased dynamics of the flexible loops in the transmembrane domain

The extramembrane loops between helices are usually less characterized in solute transporters. The HDX-MS study indicated that most periplasmic loops exhibit increased dynamics in the presence of melibiose in the absence or presence of Na^+^ (Figs. 5 ▸, 6 ▸*b* and 6 ▸*c*). The periplasmic loops at the interface between the two domains, including loop_1–2_, loop_11–12_ and loop_7–8_, also show increased deuterium uptake in the presence of both melibiose and Na^+^. The cytoplasmic loop between the shortest transmembrane helix VIII and helix IX contains a short helix that runs parallel to the membrane plane, called ICH2. This region contacts the N-terminal helix V, sealing the gate at the outward-facing conformation to prevent solvent from accessing the cytoplasmic salt-bridge network (Hariharan *et al.*, 2026 ▸). The increased flexibility at the cytoplasmic ICH2 and periplasmic loop_1–2_, loop_11–12_ and loop_7–8_ could be interpreted as a tendency to form a transition-competent conformation, with closing at the periplasmic gate and opening at the cytoplasmic gate.

## 7. Molecular mechanism of substrate translocation across the membrane

### 7.1. Sugar translocation

The molecular mechanisms underlying the observed positive cooperativity in sugar–cation binding are fascinating. Sugar affinity is coupled to protein conformational transition, which is allosterically modulated by the co-substrate cation. When the protein opens its binding site to the periplasmic side, the increased affinity by Na^+^ leads to binding of galactoside at lower concentrations. When the sugar-bound protein opens its cavity to the cytoplasm, the reduced sugar-binding affinity facilitates sugar release into the cytoplasm and prevents rebinding, which is favored when the concentration is higher than that outside the membrane in the active-transport mode.

### 7.2. Na^+^ translocation

Because Na^+^ affinity does not change significantly between the outward- and inward-facing states, the *K*_d_ value for Na^+^ binding in the inward-facing state is less than 1 m*M*, which is lower than the intracellular Na^+^ concentration maintained at 3–5 m*M* (Schultz & Solomon, 1961 ▸). The question is: what is the mechanism for the intracellular Na^+^ release? Previous mutagenesis showed an all-or-none effect of single-site mutations at either Asp55 (Hariharan, Bakhtiiari * et al.*, 2024 ▸) or Thr121 (Katsube *et al.*, 2022 ▸). For example, the D55A mutant eliminated Na^+^ binding in MelB_St_ (Granell *et al.*, 2020; Katsube *et al.*, 2022 ▸), suggesting that Na^+^ coordination requires precise positioning of each side chain within this pocket. It seems that the movement of the Asp55 side-chain rotamers after sugar release could displace the bound Na^+^ from the pocket to the empty internal cavity. Na^+^ in the cavity should favor rebinding in the absence of membrane potential, given the higher intracellular Na^+^ concentration relative to the higher affinity at the binding site; however, the membrane potential (ΔΨ, inside negative) pre-established in bacterial membranes can promote Na^+^ departure from the internal cavity into the cytoplasm. This interpretation is consistent with the previous study showing that ΔΨ is required for Na^+^-coupled melibiose uptake mediated by MelB_St_ (Guan *et al.*, 2011 ▸).

## 8. Schematic illustration of MelB_St_ uniport and symport cycles

### 8.1. Uniport mechanism with D59C MelB_St_

**Figure 7:**
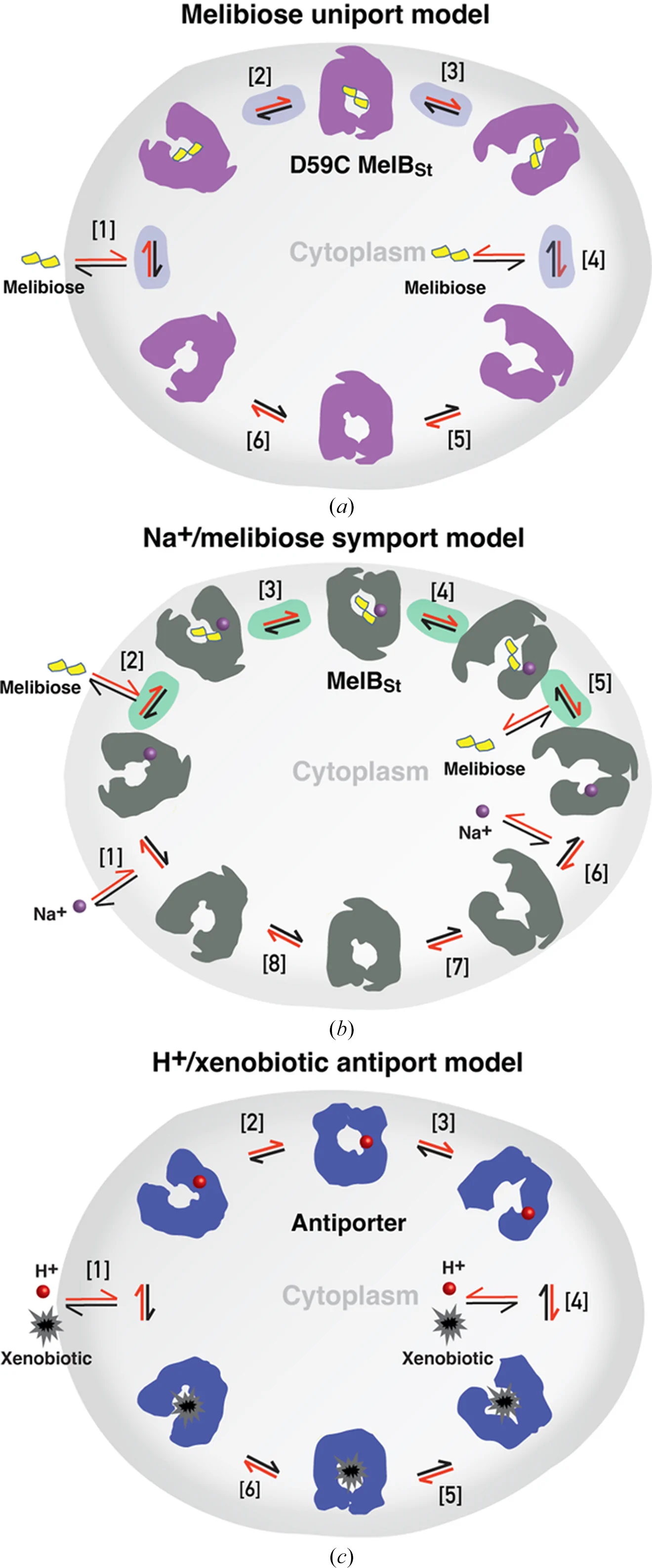
Schematic illustration of uniport, symport and antiport processes of MFS transporters. (*a*) Uniport mechanism in the D59C MelB_St_ mutant. (*b*) Na^+^/melibiose symport mechanism. The stepped-binding models are described in the main text. MelB_St_, Na^+^ and melibiose are labeled. The steps [1–4] in the uniport mode and [2–5] in the symport mode are involved in the melibiose-exchange reaction and highlighted in blue or green on the arrows, respectively. This figure was modified from Fig. 6 of Guan & Hariharan (2021 ▸). (*c*) H^+^/xenobiotics antiport mechanism. Xenobiotics and H^+^ are labeled. This anticooperativity-based ‘ping-pong’ exchange mechanism is described in the main text.

A single D59C mutation allows MelB_St_ to function as a uniporter instead of a symporter. The transport involves six steps (Fig. 7 ▸*a*), starting at step [1], with the cycle proceeding clockwise as shown by the red arrows. Melibiose recognition occurs when the apo D59C mutant adopts its outward-facing conformation, triggering alternating-access conformational changes and shifting MelB_St_ into an occluded state [2]. The cavity then opens toward the cytoplasm [3], releasing melibiose into the cell [4]. MelB_St_ subsequently closes the cytoplasmic gate [5] and returns to the outward-open state [6], ready for another cycle. Since the *K*_d_ for melibiose binding to the D59C mutant is about 6 m*M* (Guan & Hariharan, 2021 ▸), this process operates only at high environmental melibiose levels, with no intracellular accumulation. For instance, in a melibiose fermentation assay on MacConkey agar, 30 m*M* melibiose and α-galactosidase are needed. This enzyme quickly hydrolyzes incoming melibiose into glucose and galactose, preventing its buildup inside the cell and enabling downhill transport. At step [4], melibiose release or rebound depends on the intracellular concentration. Due to hydrolysis by α-galactosidase, cytoplasmic melibiose remains low, favoring release over rebound during fermentation. Transport of α-NPG by this uniport also resulted from intracellular α-galactosidase that breaks it down into *p*-nitrophenol and galactose, rapidly clearing it from the cytoplasm (Hariharan *et al.*, 2026 ▸). In active-transport assays performed at 0.4 m*M* melibiose, no uptake was detected because this concentration is ten times lower than the binding affinity of this mutant, and more importantly, the coupling to the membrane potential is lost due to a lack of cation binding.

The uniporter D59C MelB_St_ can also mediate reversible translocation, as demonstrated by the melibiose-exchange reaction (Ethayathulla *et al.*, 2014 ▸; Guan & Hariharan, 2021 ▸). In contrast to the downhill influx, the outward-directed translocation begins at step [4] and proceeds in an anti­clockwise direction to step [3], as shown by the black arrows, then oscillates among steps [1], [2], [3] and [4]. The encounter–exchange reaction involves only these four steps, as emphasized by the blue highlights on the arrows.

Overall, the uniport mode appears to be the fundamental function of a solute transporter. The role of cation binding and co-translocation constitutes a detachable regulatory component that can be excised without compromising the primary uniport mechanism.

### 8.2. Na^+^/melibiose symport mechanism with MelB_St_

For the symport process, an eight-step model, including two additional steps, cation binding and release, was constructed, and a stepped-binding mechanism was proposed (Fig. 7 ▸*b*). Active transport in the symport reaction begins with Na^+^ binding in step [1], followed by melibiose binding in step [2], and then proceeds in a clockwise direction around the cycle, as indicated by the red arrows. This sequential binding model is supported by the finding that cation binding, especially Na^+^ binding, cooperatively increases melibiose-binding affinity (Hariharan & Guan, 2017 ▸) and stabilizes the outward-facing state (Hariharan *et al.*, 2026 ▸). After the conformational change in step [3] to an occluded intermediate and in step [4] to the inward-facing state, melibiose release occurs in step [5]. This results from a substantial decrease in sugar-binding affinity when the inner barrier is broken (Hariharan, Shi *et al.*, 2024 ▸). This reduced affinity facilitates melibiose release and prevents its rebound, a critical step for the active-transport process to build up substrate concentrations relative to the extracellular concentration. Na^+^ release occurs at step [6]. In the presence of an electrochemical Na^+^ gradient, cytoplasmic release of the bound cation is favored, which should facilitate cytoplasmic closure and prevent melibiose rebound. In the absence of a membrane potential, Na^+^ release is slow because the intracellular Na^+^ concentration exceeds the binding affinity in the inward-facing state. After both substrates have been released, the empty carrier returns to the favored outward-facing state through steps [7–8].

Symporters are also engaged in an outward-directed efflux; it begins at step [6] and proceeds counterclockwise around the full circle, as indicated by black arrows. For the encounter–exchange reaction, it involves the same four steps as a uniporter, highlighted in green on the arrows (Fig. 7 ▸*b*), but in the symporter, MelB_St_ is bound by a cation.

Notably, the symport modes are similar to the H^+^-coupled lactose permease LacY (Guan & Kaback, 2006 ▸). In MelB, the role of coupling Na^+^ was clearly delineated. Kinetically, previous studies showed that Na^+^ binding and co-transport greatly improves the transport *K*_m_ compared with the H^+^-coupled mode (Niiya *et al.*, 1980 ▸; Bassilana *et al.*, 1987 ▸; Jakkula & Guan, 2012 ▸). Structurally and dynamically, the coupled Na^+^ was shown to act as an allosteric activator (Hariharan *et al.*, 2026 ▸; Liang & Guan, 2024 ▸). Unlike in receptor systems, this allosteric activator Na^+^ is also co-transported with the primary substrate melibiose.

## 9. Model for H^+^/drug antiport cycles

MFS transporters also have cation-coupled antiporters. An example is the well studied H^+^-coupled multiple-drug transporter MdfA from *E. coli* (Edgar & Bibi, 1999 ▸; Bibi *et al.*, 2001 ▸; Fluman *et al.*, 2012 ▸). This MFS antiporter utilizes the electrochemical H^+^ gradient to confer bacterial resistance by extruding toxic compounds, including lipophilic cations and neutral antibiotics. A simple transport model based on anti­cooperativity for antiport process was proposed (Fig. 7 ▸*c*). In MdfA, substrates and protons compete for binding to MdfA, and competitive inhibition between the primary substrates and protonation has been established (Fluman *et al.*, 2012 ▸). Thus, the substrate-binding affinity is competitively inhibited by protonation, and protonation is also inhibited by the binding of xenobiotics. Therefore, a ‘ping-pong’ exchange mechanism is proposed (Fig. 7 ▸*c*). The antiporter is protonated in the outward-facing conformation [1]; the protonated form shifts to the intermediate state [2] and then the inward-facing state [3]. Binding of the primary substrate from the cytosol or inner leaflet to the inward-open cavity should decrease the H^+^ affinity and facilitate deprotonation [4]; the substrate-bound antiporter shifts its conformation to the occluded intermediate state [5] and then the outward-facing conformation [6]. The favored protonation processing in the outward-facing conformation should decrease the affinity of the primary substrate and facilitate its release to the periplasmic space.

Unlike the symporter, where the transporter carries the empty or both substates fully loaded binding sites during the conformational transitions between the inward- and outward-facing conformations, the antiporter carries either primary substrate or the coupling cation to perform the conformational transition between the two states. Further investigations and more data are still needed to verify and explain this proposed anticooperativity-based antiport mechanism in greater detail.

## 10. Summary and remarks

Recent structural, functional and dynamic research on the bacterial melibiose permease MelB has revealed significant insights into the symport mechanisms of Na^+^-coupled MFS transporters. These include key members involved in health and disease, such as MFSD2A at major organ barriers such as the blood–brain barrier (Nguyen *et al.*, 2014 ▸; Cater *et al.*, 2021 ▸; Wood *et al.*, 2021 ▸; Chua *et al.*, 2023 ▸). However, many mechanistic questions are still unresolved, and the molecular details of how the central regulatory protein IIA^Glc^ controls MelB activity remain incomplete. Additional research is necessary to deepen our understanding of secondary transporters in physiology, disease and therapy.
